# Sustained Bulbar and Respiratory Function in a Case Most Consistent With Bulbar-Onset Amyotrophic Lateral Sclerosis Following Axial Spinal Traction: A 21-Month Report

**DOI:** 10.7759/cureus.92341

**Published:** 2025-09-15

**Authors:** Huan-Wei Chen

**Affiliations:** 1 Chiropractic, Private Chiropractic Practice, Vancouver, CAN

**Keywords:** als therapy, amyotrophic lateral sclerosis, atypical progression, axial spinal traction, bulbar-onset als, cerebrospinal fluid dynamics, functional preservation, glymphatic system, neurodegeneration, sleep disturbance

## Abstract

Bulbar-onset amyotrophic lateral sclerosis (ALS) is a rapidly progressive neurodegenerative disease characterized by early decline in speech, swallowing, and respiratory function, and is associated with a poor prognosis. We report the case of a woman whose treating neurologist determined, eight months after symptom onset, that her presentation was most consistent with bulbar-onset ALS. At 16 months, she began receiving intermittent pelvis-stabilized axial spinal traction (PSAST) as a supportive intervention. Remarkably, over the subsequent 21 months, she maintained oral intake and preserved respiratory function, an outcome atypical of the expected trajectory of bulbar-onset ALS. While this single case relies on provider reports for diagnostic confirmation, it raises the hypothesis that axial spinal traction may help sustain bulbar and respiratory function in ALS. Given the lack of effective treatment options, we present this case to raise awareness of a potential supportive approach that warrants further investigation.

## Introduction

Amyotrophic lateral sclerosis (ALS) is a progressive neurodegenerative disorder characterized by the loss of upper and lower motor neurons (UMN and LMN), leading to weakness, paralysis, and eventual respiratory failure. Bulbar-onset ALS, which accounts for nearly one-third of cases [1], manifests with early deficits in speech, swallowing, and respiratory function and is associated with a poorer prognosis, with a median survival of approximately 24 months from symptom onset [2].

Currently available pharmacological therapies, including riluzole and edaravone, provide only modest benefit in slowing disease progression, and no treatment has been shown to reverse functional decline [1]. Supportive strategies such as nutritional management, non-invasive ventilation, and physical therapy can improve quality of life but have not been demonstrated to preserve bulbar or respiratory function over extended periods.

We report the case of a woman who was diagnosed by her treating neurologist, eight months after symptom onset, with clinical features most consistent with bulbar-onset ALS. Sixteen months after symptom onset, she began intermittent treatment with a pelvis-stabilized axial spinal traction (PSAST) method. Over the following 21 months, she maintained oral intake and preserved respiratory function, outcomes notably atypical for the expected progression of bulbar-onset ALS. This, to the best of our knowledge, represents the first documented case of ALS managed with axial spinal traction, highlighting a potential supportive approach that may merit further investigation.

A speculative hypothesis is that PSAST may act through transient spinal elongation and stretching of the cerebrospinal meninges, potentially generating cerebrospinal fluid (CSF) pressure gradients that enhance glymphatic flow and facilitate the clearance of neurotoxic waste. In ALS, impaired respiratory function combined with poor sleep may further compromise CSF dynamics and glymphatic clearance [3]. Sleep disturbances are highly prevalent in ALS and substantially increase the burden of disease for both patients and caregivers [4]. In this case, the observed resolution of insomnia during frequent PSAST treatment raises the possibility that PSAST could contribute to improved sleep, although this remains conjectural and requires objective investigation.

Given the irreversible nature of motor neuron loss in ALS, sustained bulbar and respiratory function in this case coincided with the application of PSAST. Although encouraging, this single observation does not establish efficacy and should be regarded as hypothesis-generating, warranting further study.

## Case presentation

Diagnostic evaluation (summarized from provider reports)

**Figure 1:**
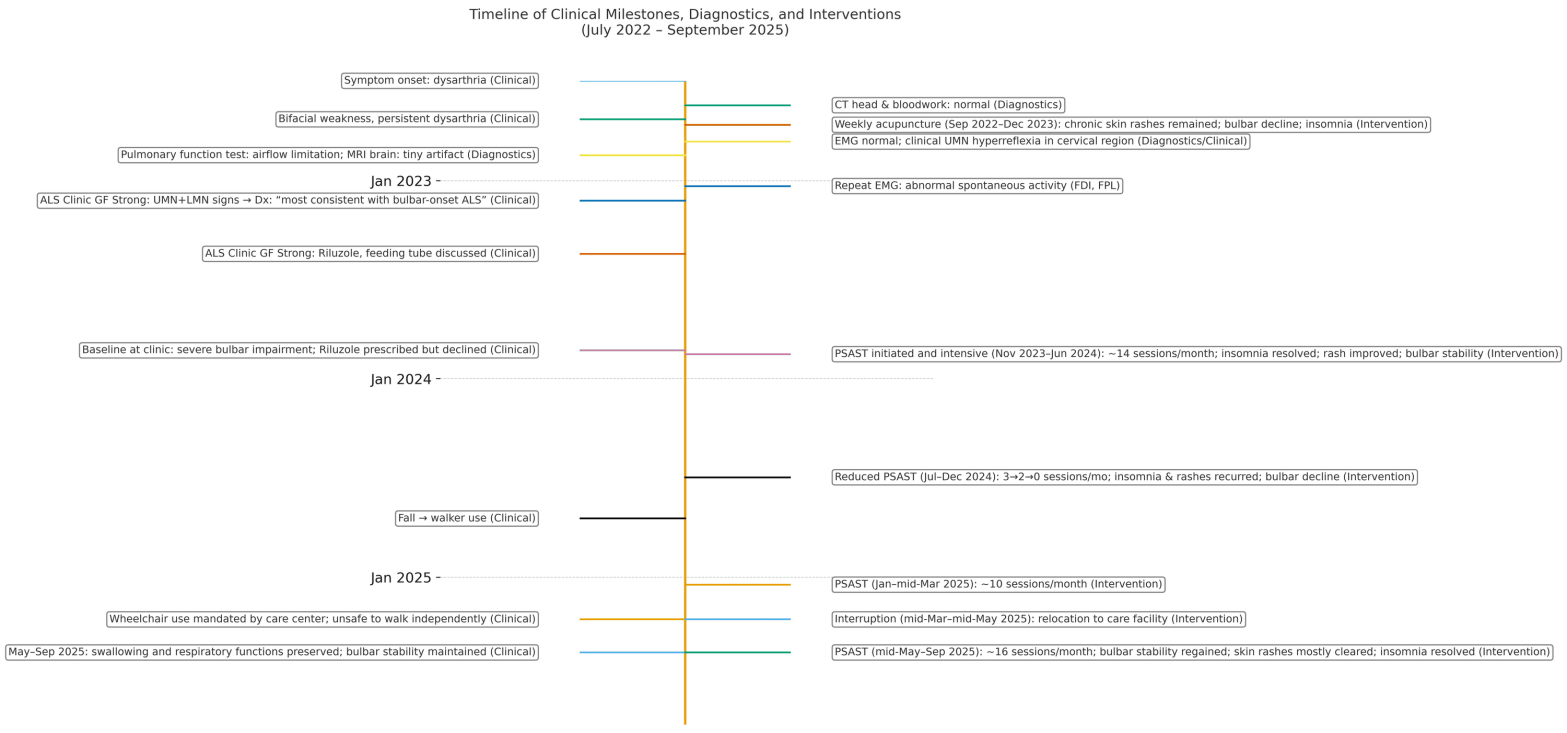
Timeline of clinical milestones, diagnostics, and interventions EMG: electromyography; ALS: amyotrophic lateral sclerosis; FDI: first dorsal interosseous; FPL: flexor pollicis longus; PSAST: pelvis-stabilized axial spinal traction;

A 52-year-old right-handed woman developed sudden-onset dysarthria in July 2022 but did not seek medical attention at that time, continuing to work full-time until October 2022, by her own report. All subsequent findings are excerpted or summarized directly from provider documentation (Figure 1).

In August, a diagnostic evaluation at Vancouver General Hospital included a computed tomography (CT) scan of the head and routine blood tests, which were reported as normal. By September, her symptoms had progressed to bifacial weakness and persistent dysarthria. Examination documented brisk reflexes on the right side.

On October 21, 2022, electromyography (EMG) was performed for worsening dysarthria and new-onset liquid dysphagia. On examination, deep tendon reflexes were brisk in the right biceps and triceps, Hoffmann’s sign was positive on the right, and a diffuse rash was present on all extremities. The provider’s report indicated normal EMG findings across the bulbar, cervical, thoracic, and lumbosacral segments but interpreted the overall results as suggestive of lower brainstem involvement.

Pulmonary function testing in November 2022 demonstrated moderate airflow limitation (forced expiratory volume in 1 second (FEV₁) 1.5 L, 69% predicted; forced vital capacity (FVC) 95% predicted), although the study was incomplete due to dyspnea. A brain MRI performed during the same month showed a very small blooming artifact in the inferior right cerebellar hemisphere, with no abnormal enhancement elsewhere.

A repeat EMG performed on January 11, 2023, showed abnormal spontaneous activity in the right first dorsal interosseous (FDI) and flexor pollicis longus (FPL) muscles. At the ALS Clinic, G.F. Strong Rehabilitation Centre, on February 7, 2023, a subsequent EMG of the tongue, FDI, extensor indicis proprius (EIP), and thoracic paraspinal muscles was reported as normal. The treating neurologist documented no family history of neuromuscular disease. On the same day, he noted clinical evidence of both UMN and LMN involvement in the bulbar region and concluded that the presentation was most consistent with bulbar-onset ALS, which he reviewed with the patient.

Baseline clinical presentation (November 2023)

Sixteen months after symptom onset, the patient was living at her brother’s home, approximately 900 meters from the clinic, and was able to walk to her appointments. She reported progressive insomnia over the preceding year, stating, “I could not sleep at all,” and described longstanding skin rashes on all extremities, present for more than three years. She also noted a subjective sensation that her tongue was becoming shorter over time. On examination, tongue protrusion was limited to the level of the teeth.

For more than a year before presentation, she had received weekly acupuncture for “ALS, shortness of breath, dermatitis, throat issues, and tongue atrophy causing difficulty speaking.” During that time, her rashes remained unchanged, while dysarthria and dysphagia gradually worsened. Acupuncture was discontinued in December 2023, one month after PSAST initiation (Figure 1).

ALS Functional Rating Scale-Revised (ALSFRS-R) [5] scores (Table 1) indicated marked bulbar impairment, with nonfunctional speech (score = 0) and swallowing limited to soft foods (score = 2). According to the treating neurologist’s report, discussion of feeding tube placement began on May 16, 2023, a procedure generally recommended in ALS to maintain nutrition and reduce aspiration risk as symptoms progress. At her initial presentation to our clinic in November 2023, however, the patient remained strongly motivated to defer this intervention. Preserving bulbar function was therefore prioritized in her care plan. She was prescribed riluzole but declined to initiate therapy. Neurological examination demonstrated brisk deep tendon reflexes (3+/4) in the right upper extremity, consistent with focal UMN involvement. Given the early bulbar deficits in conjunction with these UMN signs, PSAST was initiated with the aim of supporting and potentially preserving bulbar motor pathways, as reflected in subsequent ALSFRS-R assessments (Table 1).

**Table 1:** Longitudinal trajectory of Amyotrophic Lateral Sclerosis Functional Rating Scale – Revised (ALSFRS-R) total and sub-scores across 21 months of pelvis-stabilized axial spinal traction (PSAST) therapy (symptom onset July 2022; therapy initiation November 17, 2023). The ALSFRS-R is a standardized 12-item scale that evaluates bulbar, fine motor, gross motor, and respiratory function. Each item is scored from 0 (severe impairment) to 4 (normal), with a maximum total of 48. Higher scores indicate better functional status.

Time since symptom onset: Month 1 = July 2022	Month 17	Month 24	Month 31	Month 39
ALSFRS-R	11/2023	06/2024	01/2025	09/2025
Speech	0	1	0	1
Salivation	3	3	3	3
Swallowing	2	3	2	3
Handwriting	3	3	3	3
Cutting food and handling utensils	3	3	3	3
Dressing and hygiene	3	3	2	1
Turning in bed and adjusting bedclothes	3	3	2	2
Walking	4	3	2	2
Climbing stairs	3	3	1	1
Dyspnea	3	3	3	3
Orthopnea	3	3	3	3
Respiratory insufficiency	4	4	4	4
Total	34	35	28	29

Intervention and atypical progression

PSAST was initiated on November 17, 2023, as a supportive intervention aimed at slowing neurological decline (Figure 1). The treatment was provided on a compassionate, exploratory basis and at no cost. Written informed consent was obtained, with the patient acknowledging its experimental nature and the lack of guaranteed benefit.

Phase 1: Initial Intensive Treatment (Months 17 to 24, i.e., November 2023 to June 2024)

During this phase, the patient received frequent PSAST, averaging 14 sessions per month, with a brief reduction in January 2024. Within two weeks, she reported systemic improvements, including easier breathing and resolution of longstanding insomnia. By the end of the first month, chronic skin rashes showed visible healing. Bulbar function gradually improved, with ALSFRS-R scores increasing from 0 to 1 for speech and from 2 to 3 for swallowing (Table 1). Tongue protrusion improved to 10-15 mm beyond the teeth line, compared with none at baseline, suggesting preservation and possible enhancement of bulbar motor pathways. Body weight, oral intake, and respiratory function were maintained. Occasional episodes of heavy menstrual bleeding contributed to dizziness and transient limb weakness. Neurological examination continued to demonstrate brisk deep tendon reflexes (3+/4) in the right upper extremity.

Phase 2: Reduced Treatment Frequency and Clinical Decline (Months 25 to 30, i.e., July 2024 to December 2024)

Treatment frequency declined owing to multiple health issues, including a kidney infection in July, persistent heavy menstrual bleeding, and recurrent respiratory infections. Despite no-cost care and flexible scheduling, attendance remained limited, as these complications, together with logistical barriers and occasional reluctance, interfered with treatment consistency. Home-based sessions were not implemented initially because of professional boundary considerations.

Between July and September, treatment frequency declined to three sessions per month, followed by two in October and none in November or December. A fall in September reduced mobility, and by October, the patient required a four-wheeled walker. During this lapse, bulbar symptoms worsened with increased choking and greater difficulty eating. Insomnia recurred, and chronic skin rashes reappeared. By January 2025, ALSFRS-R scores indicated clinical decline (Table 1), and neurological examination showed brisk deep tendon reflexes (3+/4) in all extremities, consistent with generalized UMN involvement. These findings suggest reversion to the expected natural disease course during treatment interruption.

Phase 3: Resumed Traction (Home-Based and Clinic-Based) and Functional Maintenance (Months 31 to 39, i.e., January 2025 to September 2025)

In January 2025, PSAST was resumed at the patient’s residence in response to reduced clinic access and signs of disease progression. A modified portable massage table was installed, and sessions were conducted with family awareness while maintaining professional boundaries. Over the next 2.5 months, treatment averaged approximately 10 sessions per month.

From mid-March to mid-May, therapy was interrupted during relocation to a care facility, where wheelchair use was mandated. Treatment resumed in mid-May at the clinic, averaging 16 sessions per month, with transportation provided through HandyDART, a government-funded paratransit service. During this period, consistent therapy was associated with stabilization of bulbar function and modest clinical improvement.

By September 2025, ALSFRS-R scores indicated regained stability, with speech and swallowing subscores of 1 and 3, respectively (Table 1). Tongue protrusion remained stable at 10-15 mm, and body weight, oral intake, and respiratory function were maintained. The patient was now wheelchair-dependent, with limb strength decreased relative to January 2025, more pronounced on the right side but without significant atrophy. Some self-care ability was preserved. Insomnia had resolved, and skin rashes had cleared. Neurological examination continued to demonstrate brisk deep tendon reflexes (3+/4) in all extremities.

ALSFRS-R outcome measurements

Functional status was evaluated using the ALSFRS-R, encompassing bulbar, motor, and respiratory domains. Longitudinal changes in total and subscores across the 21-month treatment period are summarized in Table 1.

PSAST protocol

**Figure 2:**
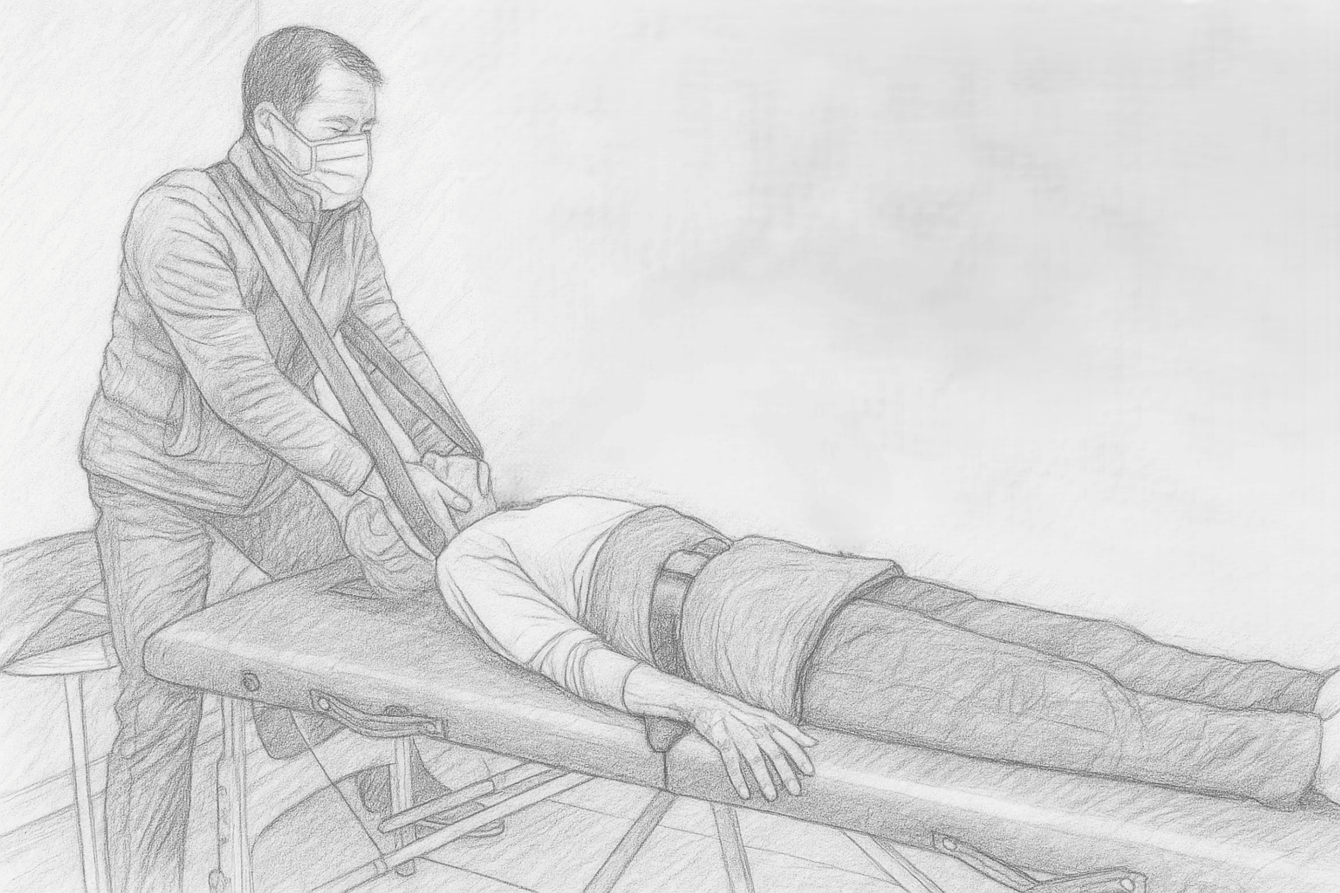
Pelvis-stabilized axial spinal traction (PSAST) demonstrated on a model using a modified portable massage table. The configuration incorporates a pelvic safety belt for stabilization and a strap anchored across the practitioner’s upper back to reinforce head support, enabling controlled axial spinal traction. Image credit: Author (as practitioner) and consenting model; original photograph digitally modified into a pencil drawing.

PSAST is a manually delivered, long-axis spinal traction technique. The patient lies supine with the pelvis secured by a safety belt, while a strap anchored across the practitioner’s upper back supports the head and stabilizes hand placement. This configuration allows controlled traction through mechanical leverage and efficient force transfer, thereby reducing reliance on direct arm exertion and minimizing practitioner strain (Figure 2). A brief, regulated pull is applied along the spine’s long axis, longer than a thrust but shorter than sustained traction, resulting in palpable cervical elongation. The applied force is individualized according to body type, cervical flexibility, and tolerance, beginning at low intensity and increasing gradually as tolerated.

PSAST employs simple, low-cost equipment that enables stable pelvic anchoring and precise head control. In principle, with structured caregiver training and continuous professional oversight, the method might be adaptable for home-based application in selected ALS patients. However, unsupervised use is not recommended due to potential safety concerns.

## Discussion

Safety and study limitations

Biomechanical studies support the cervical spine’s tensile tolerance. Van Ee et al. reported ligamentous failure thresholds of approximately 2400 N in the upper cervical spine and 1800 N in lower segments, with active musculature increasing tolerance to over 4000 N [6]. These findings provide context for the safety of conservative axial spinal traction. In this case, PSAST was administered with careful pelvic stabilization, gradual force progression, and continuous patient feedback, with no adverse events observed.

In the context of progressive neurological conditions such as ALS, safety extends beyond avoiding immediate harm to ensuring that patients do not delay or forgo established standard-of-care therapies. Pharmacological agents such as riluzole and edaravone provide modest but proven benefits [1] and remain essential components of ALS management. PSAST should therefore be regarded strictly as a supportive adjunct to, and not a replacement for, established treatment protocols.

A key limitation of this case is diagnostic uncertainty. The available EMG findings did not fulfill formal El Escorial [7] or Awaji criteria [8] for ALS. The diagnosis, “most consistent with bulbar-onset ALS,” as documented by the treating neurologist, was based on clinical impression rather than on complete exclusion of alternative diagnoses. This report follows the patient’s care as documented by the treating neurologist.

As a single case report, these findings do not establish efficacy and cannot support statistical inference. They should not be interpreted as a therapeutic recommendation. Instead, they are exploratory and hypothesis-generating. Sharing such anecdotal observations may encourage additional case documentation and independent evaluation.

Functional outcomes, systemic improvement, and a proposed glymphatic mechanism

In this case, functional outcomes appeared to coincide with treatment frequency, with greater stability observed during periods of consistent PSAST use and clinical decline during lapses. A notable motor decline occurred during a six-month interval when traction frequency was reduced (11 sessions between July and December 2024), which may reflect the natural course of disease progression. By January 2025, all extremities demonstrated hyperreflexia (3+/4). This interval also coincided with several health complications, including recurrent infections, persistent heavy menstrual bleeding, and a fall in September; however, these factors do not account for the emergence of UMN signs. Following the resumption of PSAST, mild improvements were observed (e.g., speech improved from 0 to 1, swallowing from 2 to 3). These modest gains likely reflect the coarse scaling of the ALSFRS-R, in which subtle clinical changes are recorded as 1-point increases. Nonetheless, recovery was partial and did not restore the higher functional level documented earlier.

The patient’s chronic skin rashes improved during periods of frequent PSAST and recurred during lapses. The mechanism underlying this temporal association is unclear. Neuroimmune and autonomic factors are recognized contributors to eczema [9], but any involvement of CSF or glymphatic dynamics remains speculative and is not supported by current evidence.

PSAST was initiated 16 months after symptom onset, a stage at which many patients with bulbar-onset ALS are approaching feeding tube placement or respiratory compromise within the subsequent two to eight months [10]. In bulbar-onset ALS, this accelerated decline during the second year often contributes to the median survival of approximately 24 months. In the present case, however, bulbar and respiratory function were maintained for an additional 21 months beyond treatment initiation. While this atypical course may reflect phenotypic variability or diagnostic uncertainty, the duration of preserved function substantially exceeds the trajectory expected from the natural history of bulbar-onset ALS.

The glymphatic system is an astroglia-dependent clearance pathway critical for central nervous system homeostasis. It facilitates the removal of neurotoxic waste through convective exchange between CSF and interstitial fluid, with activity most pronounced during deep sleep. Impaired glymphatic function has been implicated in several neurological disorders, including Alzheimer’s disease and Parkinson’s disease, and may also contribute to toxic protein aggregation in ALS [3].

One speculative hypothesis is that PSAST-induced spinal elongation may transiently stretch the meninges and generate CSF pressure gradients. Whether such changes could influence glymphatic clearance or sleep physiology is unknown; at present, this remains conjectural and requires objective study. As noted in the limitations, this proposed mechanism is based solely on anecdotal observations rather than physiological measurements and should be regarded as hypothesis-generating only.

If such fluid dynamics occur, they might modulate glymphatic flow and influence the clearance of inflammatory mediators or neurotoxic protein aggregates. In this case, improved sleep quality was observed during periods of frequent PSAST, although no causal relationship can be inferred. Sleep disturbances are highly prevalent in ALS and significantly increase disease burden for both patients and caregivers [4]. The observed resolution of insomnia in this case may be clinically relevant. Nonetheless, any potential connection between axial spinal traction, sleep, and glymphatic function remains speculative and requires objective evaluation. Given the overlapping pathological mechanisms among several neurodegenerative diseases [11], exploratory studies extending beyond ALS could be considered.

## Conclusions

This single case describes a woman whose treating neurologist judged her presentation to be most consistent with bulbar-onset ALS, though diagnostic uncertainty remains. The observations are anecdotal, limited to a single patient, and cannot establish efficacy. Over a 21-month observation period following the initiation of PSAST, bulbar and respiratory function appeared relatively stable, and systemic symptoms such as insomnia and skin rashes improved. These findings should be regarded as hypothesis-generating only.

One speculative hypothesis is that PSAST-induced spinal elongation may transiently stretch the meninges and generate CSF pressure gradients. Whether such fluid dynamics could influence glymphatic clearance or sleep physiology remains unknown and requires objective study. These potential mechanisms are conjectural but warrant further exploration, as they may extend beyond ALS to other neurodegenerative disorders.

Although encouraging, this single observation should not be interpreted as a therapeutic recommendation. The findings are hypothesis-generating and underscore the need for additional case documentation to determine whether similar outcomes are observed. Given the very limited treatment options for ALS, we present this case to raise awareness of a potential supportive approach that may merit further investigation in all clinical settings.
